# Supplementary material for: Tumor Expression of Cyclin-Dependent Kinase 5 (Cdk5) Is a Prognostic Biomarker and Predicts Outcome of Oxaliplatin-Treated Metastatic Colorectal Cancer Patients
Source: Cancers (Basel). 2019 Oct 11;11(10):1540. doi: 10.3390/cancers11101540 (PMC6826373; doi:10.3390/cancers11101540)
Supplement: Supplementary file 1 [file cancers-11-01540-s001.pdf]

Supplementary Materials

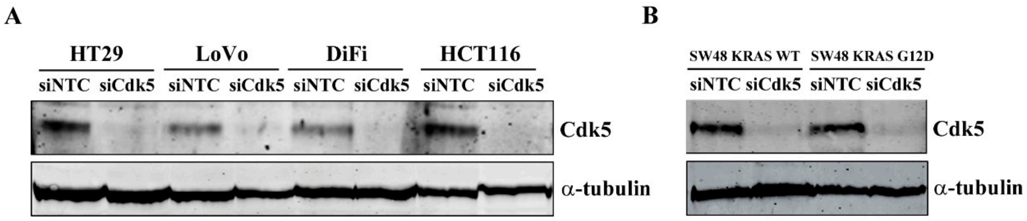

Figure S1. Cdk5 siRNA-mediated gene silencing in CRC cells. Representative western blot images showing Cdk5 protein expression after Cdk5 silencing (siCdk5) in HT29, LoVo, DiFi and HCT116 cells **(A)** and in SW48 KRAS WT and SW48 KRAS G12D cell lines **(B).** Alpha-tubulin was used as endogenous control. siNTC= control cells.

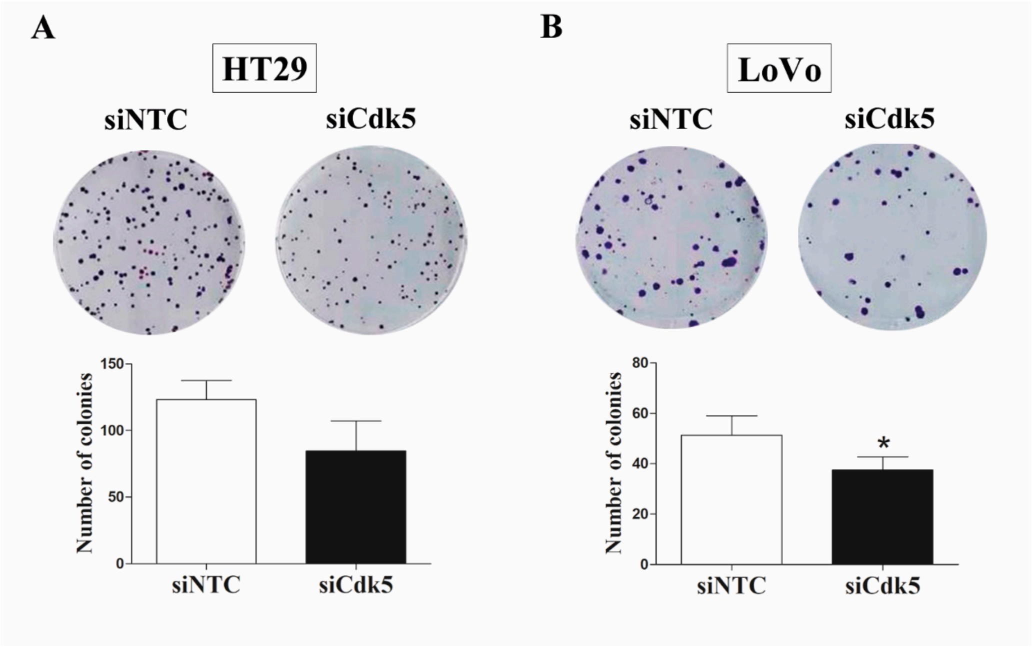

Figure S2. Effect of Cdk5 siRNA-mediated gene silencing on HCT116 cell proliferation and HT29 and LoVo colony formation. Representative images and corresponding graphs of HT29 (**A**) and LoVo (**B**) colony formation, results were obtained from at least three independent experiments. *p-value < 0.05.

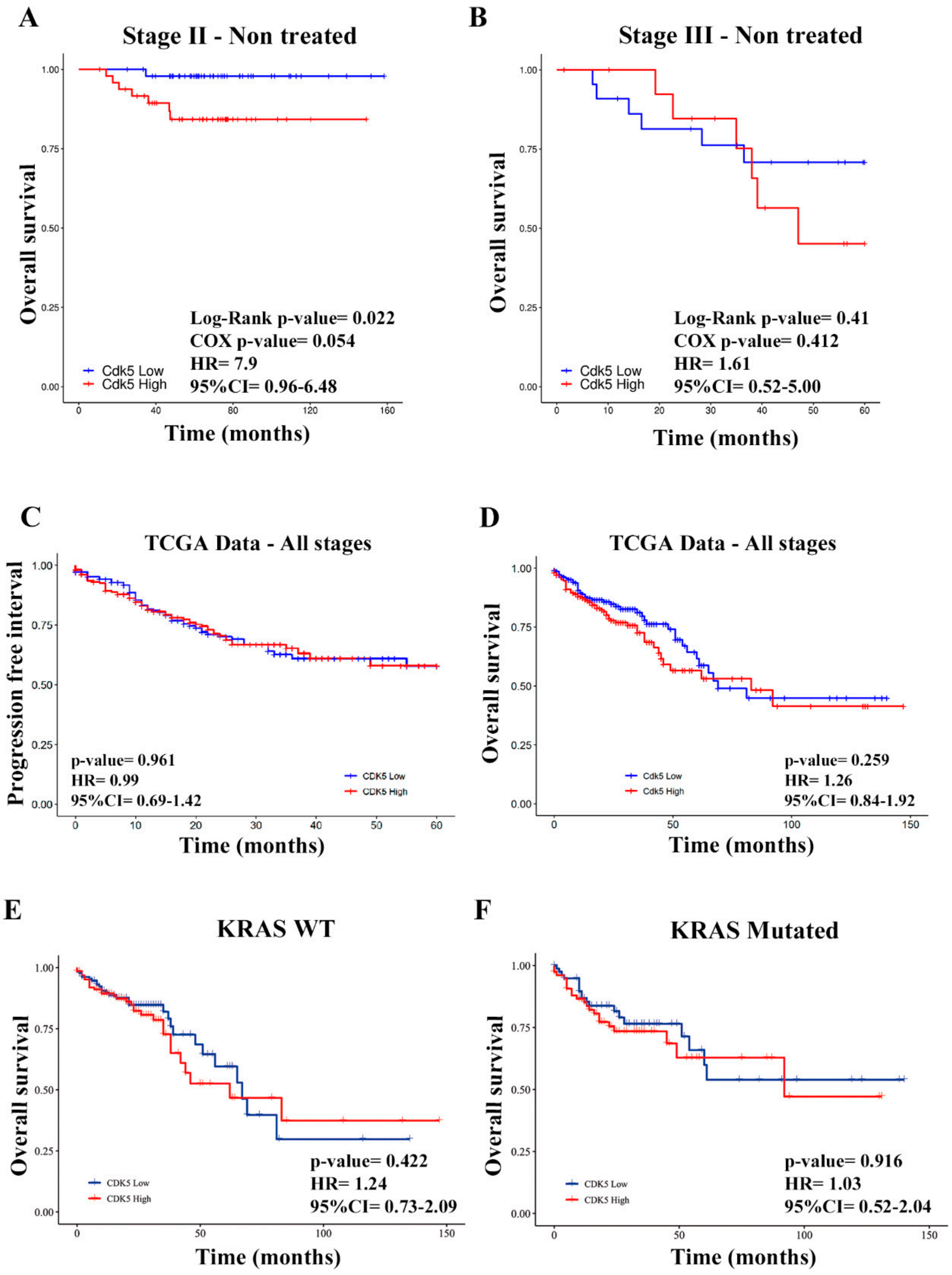

Figure S3. Kaplan-Meyer analysis of OS and PFI depending on Cdk5 expression and KRAS mutational status (**A**) OS in 98 stage II CRC patients split by the median of Cdk5 expression (cohort B). (**B**) OS in 37 non-treated stage III CRC patients split by the median of Cdk5 expression (cohort C) (**C**) PFI in the TCGA-COAD including 473 stage I-IV CRC patients split by the median of Cdk5 expression (cohort F) (**D**) OS in the TCGA-COAD including 473 stage I-IV CRC patients split by the median of Cdk5 expression (cohort F). (**E**) OS for 72 stage II CRC patients with WT Kras and split by the median of Cdk5 expression. (**F**) OS for 26 stage II CRC patients with mutated Kras and split by the median of Cdk5 expression (cohort B).

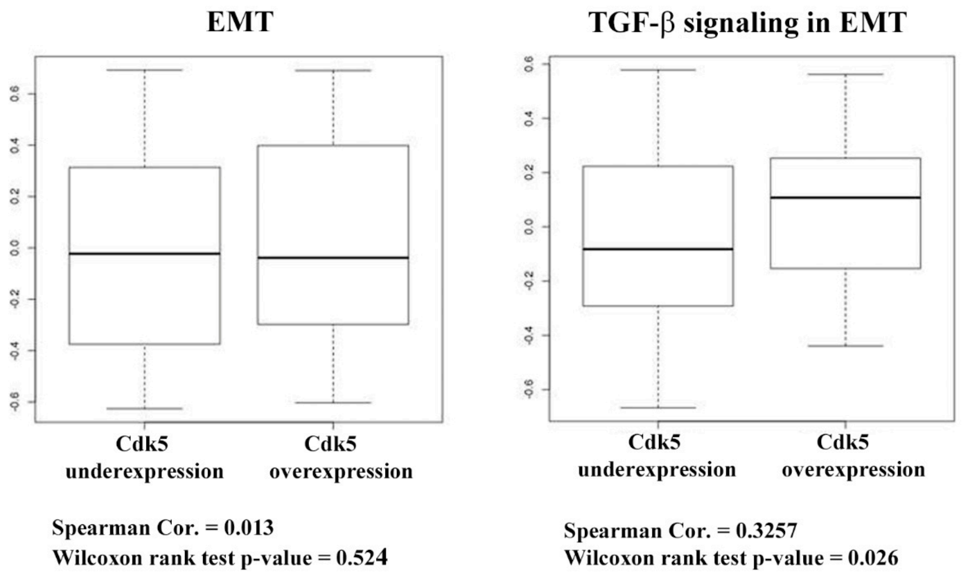

Figure S4. EMT-related enriched hallmark correlations with Cdk5 expression. Box plots representing EMT-related enriched hallmark correlations with Cdk5 expression obtained by GSEA overlap in stage II CRC patients (cohort B). The three quartiles are represented in the box, and the extreme lines represent the maximum and minimum values, while the median is represented by a thicker line.

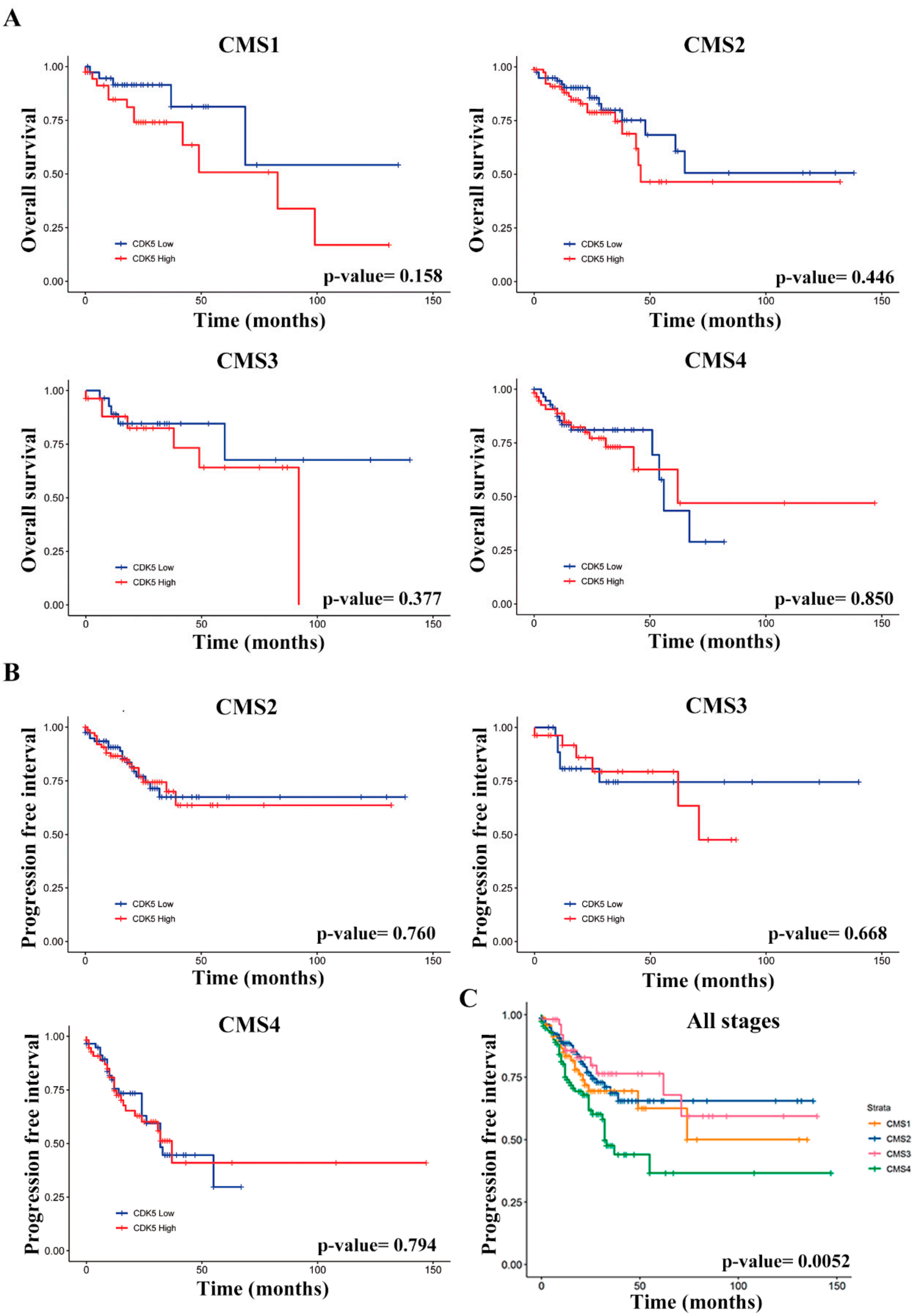

Figure S5. Kaplan–Meyer analysis of OS and PFI depending on Cdk5 expression and CMS group. (**A**) OS in the TCGA-COAD including stage I–IV CRC patients classified according to the CMS group. CMS1, n = 78; CMS2, n = 159; CMS3, n = 55; and CMS4, n = 110. In all groups, patients were split according to the median of Cdk5 expression (cohort F). (**B**) PFI in the TCGA-COAD CMS2 (n = 159), CMS3 (n = 55), and CMS4 (n = 110) subgroups; patients were split according to the median of Cdk5 expression (cohort F). (**C**) PFI in the TCGA-COAD including 473 stage I–IV CRC patients according to their classification into four CMS groups (cohort F).

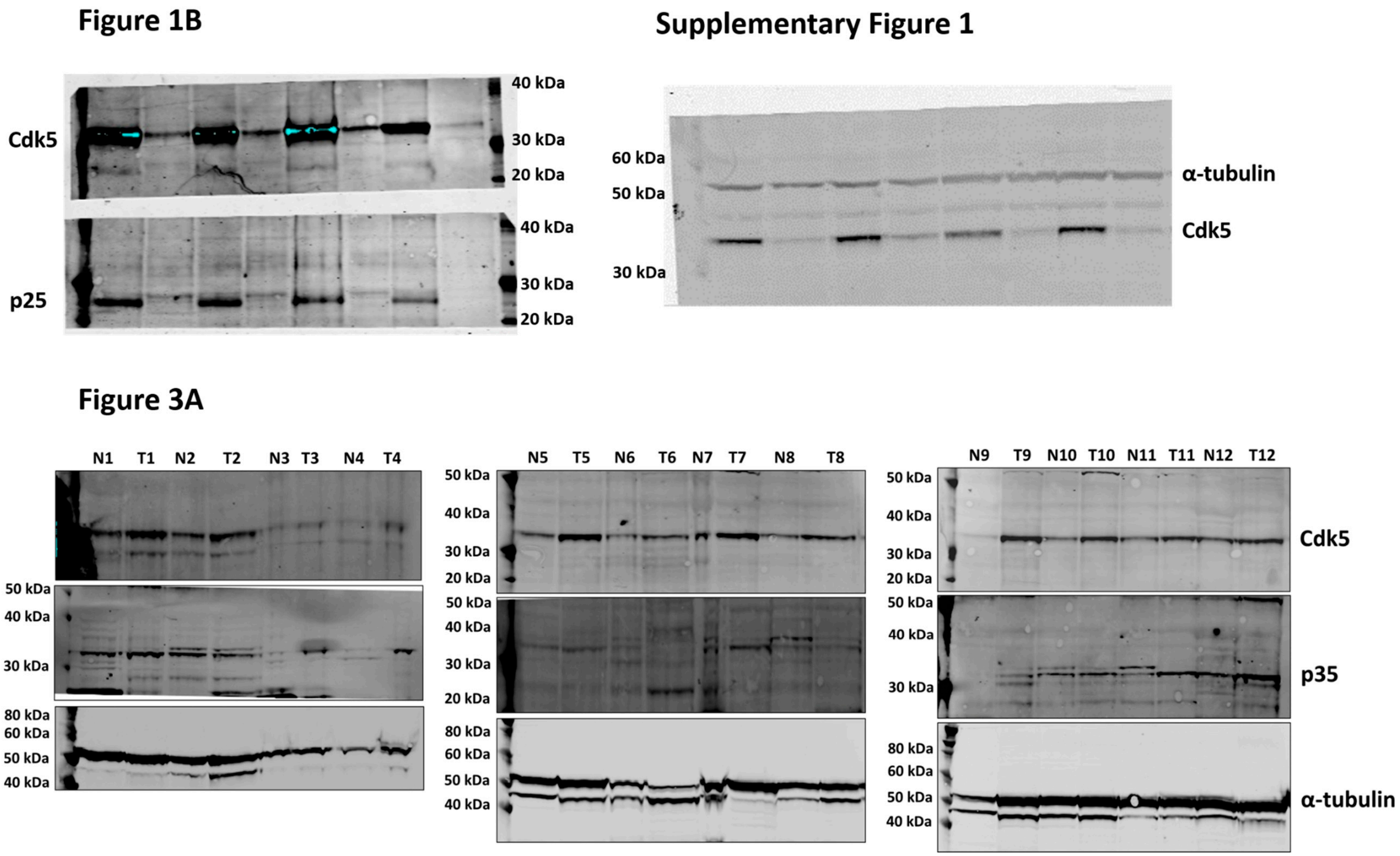

Figure S6. Images displaying non-cropped western blots from Figures 1B, 3A, and S1.
